# Public Prevention Plans to Manage Climate Change and Respiratory Allergic Diseases. Innovative Models Used in Campania Region (Italy): The Twinning Aria Implementation and the Allergy Safe Tree Decalogue

**Published:** 2019-01-06

**Authors:** V. Patella, G. Florio, D. Magliacane, A. Giuliano, L.F. Russo, V. D’Amato, V. De Luca, G. Iaccarino, M. Illario, J. Bousquet

**Affiliations:** 1 Division of Allergy and Clinical Immunology, Department of Medicine ASL Salerno, “Santa Maria della Speranza” Hospital, Battipaglia, Salerno, Italy; 2 Postgraduate Program in Allergy and Clinical Immunology–University of Naples Federico II, Naples, Italy; 3 Laboratory of Environmental Analysis, Department of Public Health, ASL Salerno, Salerno, Italy; 4 Referente Promis, ASL Salerno, Salerno, Italy; 5 Direzione Sanitaria, ASL Salerno, Italy; 6 Research and Development Unit, Federico II University Hospital, Naples, Italy; 7 Department of Advanced Biomedical Sciences, University of Naples Federico II, Naples, Italy; 8 Division for Health Innovation, Campania Region and Federico II University Hospital Naples (R&D and DISMET) Naples, Italy; 9 MACVIA-France, Fondation partenariale FMC VIA-LR, Montpellier, France; 10 VIMA, INSERM U 1168, VIMA : Ageing and chronic diseases. Epidemiological and public health approaches, Villejuif, Université Versailles St-Quentin-en-Yvelines, UMR-S 1168, Montigny le Bretonneux, France, Euforea, Brussels, Belgium, and Charité, Universitätsmedizin Berlin, Humboldt-Universität zu Berlin, and Berlin Institute of Health, Comprehensive Allergy Center, Department of Dermatology and Allergy, Berlin, Germany

**Keywords:** Allergic Rhinitis, Air pollution, Asthma, Climate Change, Health Innovation Programs, Public Health

## Abstract

In recent years, climate change has been influenced by air pollution, and this destructive combination has justifiably sounded an alarm for nations and many institutional bodies worldwide. Official reports state that the emission of greenhouse gases produced by human activity are growing, and consequently also the average temperature. The World Health Organization (WHO) believes that health effects expected in the future due to climate change will be dramatic, and has invited international groups to investigate potential remedies. A task force has been established by the Italian Society of Allergology, Asthma and Clinical Immunology (SIAAIC), with the aim to actively work on correlation between pollution and climate change. The Task Force provided prevention tools to suggest city leaders how to improve the health conditions of allergic people in public urban parks. The “Allergy Safe Tree Decalogue” suggests the preparation and maintenance of public low allergy-impact greenery. Through the Twinning ARIA project, the Division for the Promotion and Enhancement of Health Innovation Programs of Campania Region (Italy), sought to promote the implementation of the project in the regional Health System. The main objective will be to investigate the current use and usefulness of mobile phone Apps in the management of allergic respiratory disease, through Mobile Airways Sentinel networK (MASK), the Phase 3 of the ARIA initiative, based on the freely available MASK App (the Allergy Diary, Android and iOS platforms). The effects of these prevention activities will be registered and compared with monitoring efforts thanks to the Aerobiology Units, located throughout the Campania area. A joint effort between researchers and public administrations for the implementation of prevention plans coherently with the two models proposed in a specific area, i.e. the Decalogue for public administrations and the MASK Allergy Diary app for individual patients suffering from allergy, will be implemented as a pilot.

## I. INTRODUCTION

In the scientific world there are no more doubts about the correlation between global warming and atmospheric concentrations of greenhouse gases, which have increased by 30% to date since the beginning of the industrial revolution [1,2]. Most recently the Intergovernmental Committee for Climate Change reports that emissions of greenhouse gases produced by human activity are growing at an annual rate of between 0.5% and 1% [3]. With this trend, the average temperature increase is expected at approximately 4 degrees by 2100.

The World Health Organization believes that future expected health effects due to climate change, particularly those due to the progressive warming of the planet, will be amongst the most relevant health issues to be addressed in the coming decades [4].

Consequences will be relevant in the most fragile groups of the population, such as the poor, the children, the elderly and the patients suffering from chronic pulmonary disease.

## II. BRIEF DESCRIPTION AND ANALYSIS OF THE PROBLEM

Climate change is influencing the main climate variables that determine the formation of air pollutants (increase in temperature and in the rate of solar irradiation), causing a greater formation of secondary pollutants in the atmosphere, notably of the tropospheric ozone and of fine particulate matter (PM10) [5].

The effects of climate change on respiratory and allergic diseases are numerous. It is known that there is a correlation between asthma and climatic factors, such as meteorological variables, allergens and atmospheric pollutants.

Climatic variables strongly influence allergic diseases, either directly - by acting on the respiratory tract, or indirectly - by modifying the concentrations of allergens and atmospheric pollutants (Box 1).

BOX 1 **ROLE OF MAIN ENVIRONMENTAL DETERMINANTS IN RELATION TO CLIMATE CHANGE**

□ Increasing temperatures and extreme events: the increase in temperatures recorded in recent years causes the elongation and early arrival of the pollen season, an increase in pollen production, and the spread of invasive species.
□ Increase in the frequency of storms and intense rain: conditions of intense humidity and wind during thunderstorms can cause the breakage of pollen grains due to osmotic shock, promoting the release of allergenic antigens into the atmosphere (pollutant storms). Episodes of severe asthma during thunderstorms have been noted.
□ Increase in the frequency of fires and heat waves: in some regions, changes in the mean and variability of temperature and precipitation are expected to increase the frequency of fires and heat waves, with potential consequences on respiratory health.
□ Heat waves can aggravate pollution. Studies have shown that air pollution has an additional impact on mortality during a heat wave [6].
□ High concentrations of ozone are the cause of excessive mortality due to respiratory issues during a heat wave [7].
□ Changing weather conditions also influence the transportation, dispersion and depositing of atmospheric pollutants, and may impact the effect on health associated with fine powders and gaseous pollutants.
□ New atmospheric circulation models, caused by climatic variations, could also promote the long-distance transport of allergenic pollens, increasing the risk of new sensitisations among the allergic population [8].
□ Effects on environmental air quality (indoor/outdoor) of marine aerosol currents may have an influence on the increase in respiratory illnesses in the general population.

Weather-climatic determinants contribute to the increased toxicity of atmospheric pollutants, a variation in the season, the quantity and toxicity of pollen [9], an increase in allergen-inducing invasive species, and an increase in vector insects [1].

All of these factors could increase health risks in outdoor and indoor environments, and there may be an increase in allergic or asthmatic crises.

Also chronic exposure to chemical pollutants, in outdoor (particulates, ozone, nitrogen oxide, etc.) and indoor air (VOCs - Volatile Organic Compounds, ozone and particulates), has various effects on human health, and can also trigger allergic and asthmatic crises in sensitive individuals [10,11].

Climate change can influence local and regional air quality through changes in the rates of chemical reactions in the atmosphere, the heights of the mixing layers of pollutants and changes in the characteristics of air flows that regulate the transport of pollutants, influencing the development, transportation, dispersion, concentration and depositing of both chemical and biological atmospheric pollutants. Under conditions of specific atmospheric circulation, the transportation of pollutants may be greater and span large distances for prolonged periods. Exposure to air pollutants (PM, O3, NOx, DEPs - diesel exhaust particles, CO2) can interact with allergens transported by pollen, and may increase the risk of atopic sensitisation and symptoms in allergic individuals. High temperatures promote the emission of larger quantities of VOCs from forests and the soil. Precipitations promote the oxidation of SO2 (sulphur dioxide); furthermore, warmer temperatures promote microbial activity of the soil and lead to an increase in the production of NOx (nitrogen oxide), which is also a precursor of the tropospheric ozone.

In addition to allergic and respiratory diseases, several studies on mortality show an increase in cardiovascular risk derived by atmospheric pollution when associated with persistently high temperatures.

The increase in environmental temperature in warmer seasons causes an increase in atmospheric ozone levels. Ozone has irritating and toxic effects on the mucous membranes of the respiratory tract, with a consequent decrease in the respiratory function. It has been demonstrated that exposure to high concentrations of ozone is associated with an increase in hospital admissions due to asthmatic crises and other respiratory diseases [1,2] (Box 2).

BOX 2 **CLIMATE CHANGE ALSO INFLUENCES THE EXPOSURE TO POLLUTANTS IN INDOOR AIR. CONDITIONS THAT MAY OCCUR DUE TO THERMAL (HEAT AND FROST WAVES) AND METEOROLOGICAL ANOMALIES**

□ More time is spent indoors increasing the exposure period to indoor pollutants such as environmental tobacco smoke, benzene, NO2, PM and formaldehyde, which can cause an increase in the frequency of chronic respiratory symptoms, hyper-bronchial reactivity, and a reduced response to asthmatic therapy in asthmatic subjects.
□ Indoor micro-climatic alterations resulting from altered rainfall and temperature patterns have an influence on indoor biological pollutants (mould) which are important health risk factors when paired with humidity.
□ Excessive use of air conditioning during heat waves can lead to an increased exposure to microbiological contaminants which develop in the humid components of air conditioning systems.
□ Change in the ecosystem and allergenic determinants: current climate change is responsible for the early arrival of the spring pollen season, the prolongation of the flowering period and a change of the diffusion areas of main plant species.
□ The increase in the concentration of CO2 in the atmosphere and the increase in temperature (notably in the urban environment, where we are witnessing a phenomenon known as the “Urban Heat Island”, UHI) have encouraged the production of pollen and the development of certain allergenic plant species.

By virtue of these effects, climate change has an important impact on health - especially in persons affected by pollinosis - both due to the prolongation of the exposure period and to the establishment of new prevalence in the various territorial areas, with high direct and indirect socio-economic costs.

It is interesting to note that there is a close link between the socio-economic effects of climate change resulting from the choices in urban planning, energy and transport sectors that have an impact on health and well-being due to temperature increases and the amplification effect in central areas of cities (Urban Heat Island), with significant repercussions on the health of the resident population, especially the elderly and people with cardiorespiratory diseases (in particular, COPD and asthma) [12].

Climate change and high temperatures also impact social conditions, especially for the elderly and/or people with chronic diseases (e.g. COPD), due to lower attendance of public areas and meeting places (e.g. streets, squares -Urban Solitude Islands), with a high impact on the quality of life and well-being of the most vulnerable parts of the population.

## III. THE IMPORTANCE OF ADAPTATION STRATEGIES

The leaders of 190 countries around the world have gathered at various world summits to define a strategy for the substantial reduction of greenhouse gas emissions. Significant issues related to climate and health have been reported between now and 2030, 250,000 more deaths are estimated each year due to climate change. The greatest risks are related to overheating and fires, to increased malnutrition resulting from decreased food production in poor regions, to loss of working capacity in vulnerable populations; in addition, the WHO hypothesises that climate change will increase social inequalities amongst populations.

Climate change will persist for many centuries [3]; this means that we will have to cope with the impacts of climate change for at least the next 50 years. Since climate change is already under way and its consequences cannot be avoided, the reduction of greenhouse gases and other phenomena that underlie the change can only limit the speed at which the phenomenon occurs, while prevention measures can minimize potential negative consequences and prevent damages resulting from climate change.

## IV. THE ROLE OF INSTITUTIONS

The role of politics, institutions, industries and citizens can be decisive in combating climate change and limiting its consequences on health. The governance capability of local administrations as well as the degree of awareness of citizens and professionals in the sector (such as architects and urban planners) are major factors. The complexity of climate change and the related mitigation efforts require complex responses from different sectors of society [13].

The impact of climate change on health is a growing problem in Italy as well as in other Euro-Mediterranean countries. The Italian National Strategy for the Adaptation to Climate Change (SNACC) [14] defines national measures able to offer future responses to the impact of climate change in many socio-economic and natural systems, based on the assessment of sector-based vulnerabilities; the SNACC has identified a set of actions to minimise risks arising from climate change, so to increase the resilience of human and natural systems and take advantage of any opportunities arising from new climatic conditions. Priority actions and objectives for health sector are indicated in Table 1.

## V. THE “ALLERGY SAFE TREE DECALOGUE” MODEL

A popular model like the “Allergy Safe Tree Decalogue” developed by the SIAAIC (Italian Society of Allergology, Asthma and Clinical Immunology) Task Force [2] can be used as a model for primary prevention in an urban environment.

**Figure 1:**
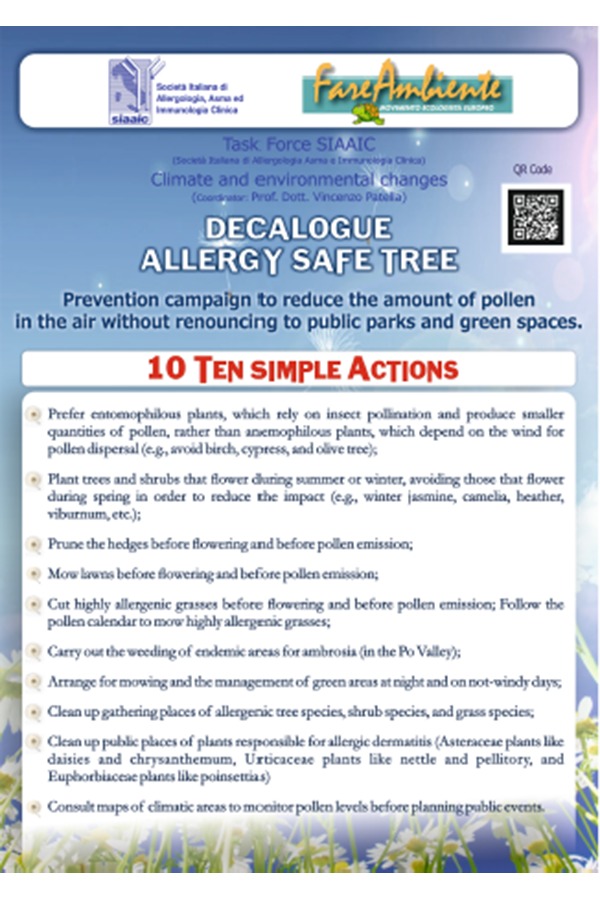
The Allergy Safe Tree Decalogue suggests that administrations that manage the public urban parks implement ten simple solutions. Favour the use of entomophilous plants, plants that have insect-led pollination and produce less pollen, and not anemophilous species that entrust the propagation of pollen to the wind (avoid plants such as: birch, cypress and olive trees) in the design of parks or in the replacement of new plants. Place trees and shrubs that bloom in summer or winter and not in spring to render the impact as minor as possible (e.g. jasmine nudiflorum, camellia, heather, liburnum). Prune hedges before flowering and before pollen emission. Mow lawns before flowering and pollen emission; follow the pollen calendar to manage graminaceous grasses that are highly allergenic. Weed endemic areas for ambrosia (Po Valley). Plan the mowing and management of greenery at night and on days with little wind. Reclaim areas of aggregation from arboreal, shrub and allergy-producing species. Reclaim public places from plants responsible for allergic dermatitis (asteraceae such as daisies and chrysanthemums, euphorbiacee plants like Christmas stars). Consult maps of climatic areas for monitoring pollen concentrations before organising public events [Patella et al. Clin Mol Allergy (2018) 16:20].

Prevention, in fact, can also take place by means of the attentive planting and care of public parks by public administrations, and of private green areas managed by private companies (condominiums, sporting centers, equipped green areas next to malls and industrial centers). In this way, the planting of allergenic decorative plants will be avoided, and the health and quality of life of “users” at risk of allergy in public areas may be improved (Figure 1).

The main aspects of the Decalogue include 10 simple suggestions to be implemented by those involved in managing public urban parks. Favour the use of entomophilous plants, plants that have insect-led pollination and produce less pollen, avoiding anemophilous species that entrust the propagation of pollen to the wind (e.g. birch, cypress and olive trees) in the design of parks or in the replacement of new plants. Place trees and shrubs that bloom in summer or winter and not in spring, to minimize their impact (e.g. jasmine nudiflorum, camellia, heather, liburnum). Prune hedges before flowering and before pollen emission. Mow lawns before flowering and pollen emission; follow the pollen calendar to manage graminaceous grasses that are highly allergenic.

Weed endemic areas for ambrosia (Po Valley). Plan the mowing and management of greenery at night and on days with little wind. Reclaim areas of aggregation from arboreal, shrub and allergy-producing species. Reclaim public places from plants responsible for allergic dermatitis (asteraceae such as daisies and chrysanthemums, euphorbiacee plants like the “*Christmas stars*”). Consult maps of climatic areas for monitoring pollen concentrations before organising public events.

The monitoring of the effects of such interventions over the years will be performed by the Aerobiology Units of R.I.M.A. (Italian Aerobiological Monitoring Network) and of INSPRA (Higher Institute for Environmental Protection and Research), located throughout the Campania Region, which will provide the air concentrations of corpuscles polluting the atmosphere.

Every week, the concentrations of different pollutants in the atmosphere are detected in the troposphere through the control units of the Regional Agency for the Environment of Campania (ARPAC) (SO2, NOx, PM10, PM2.5, lead, benzene, CO and ozone), which are important for the protection of human health, as well as the corpuscles present in the biological atmosphere (pollen, mites, mould) detected by the R.I.M.A. network. A significant reduction of these elements in the atmosphere has shown both a significant and direct correlation in the decrease of the number of hospital admissions due to respiratory and/or cardiac crises, as well as the appearance, or absence, of neoplasia in the long term.

## VI. THE MOBILE AIRWAYS SENTINEL NETWORK MODEL (MASK), THE PHASE 3 OF THE ARIA INITIATIVE (BASED ON THE FREELY AVAILABLE MASK APP)

Mobile Airways Sentinel networK (MASK), the Phase 3 of the ARIA initiative, has been initiated to reduce the global burden of rhinitis and asthma multimorbidity [15], giving the patients and the health care professionals simple tools to better prevent and manage respiratory allergic diseases. The Twinning ARIA project seeks to promote the implementation of the MASK, the Phase 3 of the ARIA initiative, in the Health System of Campania Region (which by population is the third largest in Italy); it is based on the freely available MASK App (the Allergy Diary, Android and iOS platforms). MASK is available in 16 languages and is deployed in 23 countries. The main objective will be to investigate the current use and usefulness of mobile phone Apps in the management of allergic respiratory disease.

Based on a recent study, “*The role of mobile Apps in allergic respiratory diseases: an Italian multicentre survey report*” (16), the Division for the Promotion and Enhancement of Health Innovation Programs in Campania Region intended to create a 3-phase project. The first phase, or cognitive phase, is the collection of all elements necessary to engage specialized centres that manage allergic rhinitis and asthma in Campania. Work platforms have also been identified within the regional health sector, where there is an effort to set up diagnostic and therapeutic asthma pathways (PDTA asthma) which will also use new technologies; among these is the Allergy Diary offered by the MASK project through the implementation of the Twinning ARIA. The second phase will be the development of training material for the specialised centres involved in the project, which will in turn manage the recruitment of allergic patients. The pages of the App are on the Euforea-ARIA website (www.euforea.eu/about-us/aria.html). In previous experiences, few users were allergic patients to whom the App was recommended by their physicians. Users were not requested to complete the diary for a minimum number of days. However, due to anonymisation of data, no specific information on the route of access to the App could be gathered [17,18].

The third phase is to verify how the population has improved in terms of state of health, identifying indicators such as reduced use of emergency rooms for respiratory allergy problems and a reduction the consumption of medicinal products used in the treatment of respiratory diseases.

## VII. CONCLUSIONS

Climate change alters the main climatic variables, and is directly and indirectly influenced by the presence of atmospheric pollutants. Both global warming and solar radiation contribute to the formation of secondary pollutants in the atmosphere, notably tropospheric ozone. All these factors explain the increased incidence of respiratory and allergic diseases that affect humans in different parts of the world.

Recent forecasts suggest that climate change will persist for several decades [3]. The increase in the incidence of many respiratory illnesses related to the effects of global warming on human health is likely to remain for at least the next 50 years.

Therefore, the road to be taken by institutions and inhabitants of the entire planet will be long and expensive.

The effects of climate change are already present, and their negative consequences cannot be avoided. In the general population, such phenomena can at least partly be reduced by means of loco-regional interventions, such as the use of models such as the *Allergy Safe Tree Decalogue* offered by the SIAAIC Task Force [2].

The Decalogue can be used by public administrations, as it reduces the patient’s impact to the terrible combination of the effects of climate change and air pollution. At the same time, through the Twinning ARIA project [15], involving also Campania Region, is related to MASK, i.e. the Phase 3 ARIA initiative, which acts on the disease control of the individual patient suffering from allergic respiratory illnesses. It will ensure greater results in terms of prevention as well as proof of the effectiveness of air pollution monitoring systems and their impact on primary allergic respiratory disease. A joint effort between researchers and public administrations for the implementation of prevention plans is necessary, in order to strengthen the engagement and empowerment of patients suffering from allergic rhinitis.

## 







## Figures and Tables

**TABLE 1:** PRIORITY ACTIONS TO MINIMISE RISKS ARISING FROM CLIMATE CHANGE, TO INCREASE THE RESILIENCE OF HUMAN AND NATURAL SYSTEMS AND THEIR EXPLOITATION, AND POTENTIAL OPPORTUNITIES ARISING FROM NEW CLIMATIC CONDITIONS.

ACTION	OBJECTIVE OF THE ACTION
Implementation and strengthening of health monitoring and early alert systems, including monitoring systems on water-borne diseases.	Develop initiatives and adopting tools aimed at improving knowledge (of public health operators, physicians, and other professionals, including those in non-health sectors, institutions and citizens) on risk factors related to the environment and climate change, as well as on potential prevention strategies, with special attention to respiratory diseases such as asthma and allergies.
Training and refresher courses for operators of the SSN (Sistema Sanitario Nazionale, Italian national health system).	Contribute to promoting environmental prevention policies as a consequence of new risk scenarios triggered by climate change. Improve scientific knowledge on the effects of climate change.
Strengthening of information and public communication systems.	Integrate institutions and scientific research in the health field, keeping the public opinion informed. Disseminate the use of validated tools to support management and prevention of allergic rhinitis.

## Abbreviations And Acronyms

ARIA: Allergic Rhinitis and its Impact on Asthma
SIAAIC: Italian Society of Allergology, Asthma and Clinical Immunology
MASK: Mobile Airways Sentinel networK
INSPRA: Higher Institute for Environmental Protection and Research
PM10: particulate matter
RIMA: Italian Aerobiological Monitoring Network
SNACC: The National Strategy for the Adaptation to Climate Change
VOCs: Volatile Organic Compounds, ozone and particulates
WHO: World Health Organization.

## References

[1] D’Amato G, Holgate ST, Pawankar R, Ledford DK (2015). Meteorological conditions, climate change, new emerging factors, and asthma and related allergic disorders. A statement of the World Allergy Organization. World Allergy Organ J.

[2] Patella V, Florio G, Magliacane D, Giuliano A, Crivellaro MA, Di Bartolomeo D, Genovese A, Palmieri M, Postiglione A, Ridolo E, Scaletti C, Ventura MT, Zollo A (2018). Urban air pollution and climate change: “The Decalogue: Allergy Safe Tree” for allergic and respiratory diseases care. Clin Mol Allergy.

[3] IPCC (2014). Climate Change 2014: Synthesis Report. Contribution of Working Groups I, II and III to the Fifth Assessment Report of the Intergovernmental Panel on Climate Change.

[4] Campbell-Lendrum D, Corvalán C, Neira M (2007). Global climate change: implications for international public health policy. Bull World Health Organ.

[5] Jacob DJ, Winner DA (2009). Effect of climate change on air quality. Atmos Environ.

[6] Heo S, Bell ML, Lee JT (2019). Comparison of health risks by heat wave definition: Applicability of wet-bulb globe temperature for heat wave criteria. Environ Res.

[7] Atkinson RW, Butland BK, Dimitroulopoulou C, Heal MR, Stedman JR, Carslaw N, Jarvis D, Heaviside C, Vardoulakis S, Walton H, Anderson HR (2016). Long-term exposure to ambient ozone and mortality: a quantitative systematic review and meta-analysis of evidence from cohort studies. BMJ Open.

[8] Cecchi L, Morabito M, Paola Domeneghetti M, Crisci A, Onorari M, Orlandini S (2006). Long distance transport of ragweed pollen as a potential cause of allergy in central Italy. Ann Allergy Asthma Immunol.

[9] Wolf T, Martinez GS, Cheong HK, Williams E, Menne B (2014). Protecting health from climate change in the WHO European Region. Int J Environ Res Public Health.

[10] Vallero D (2014). Fundamentals of Air Pollution Book.

[11] Viegi G, Simoni M, Scognamiglio A, Baldacci S, Pistelli F, Carrozzi L, Annesi Maesano I (2004). Indoor air pollution and aiway disease. Int j Tuberc Lung Dis.

[12] Salmond Jennifer A, Tadaki Marc, Vardoulakis Sotiris, Arbuthnott Katherine, Coutts Andrew, Demuzere Matthias, Dirks Kim N, Heaviside Clare, Lim Shanon, Macintyre Helen, McInnes Rachel N, Wheeler Benedict W (2016). Health and climate related ecosystem services provided by street trees in the urban environment. Environ Health.

[13] Misra AK (2014). Climate change and challenges of water and food security. International Journal of Sustainable Built Environment.

[14] Williams K, Gupta R, Smith I, Joyn J, Hopkins D, Bramley G, Payne C, Gregg M, Hambleton R, Bates-Brkljac N, Dunse N, Musslewhite C (2012). Suburban Neighbourhood Adaptation for a Changing Climate (SNACC) Final Report.

[15] Bousquet J, Arnavielhe S, Bedbrook A, Bewick M, Laune D, Mathieu-Dupas E, MASK study group (2018). MASK 2017: ARIA digitally-enabled, integrated, person-centred care for rhinitis and asthma multimorbidity using real-world-evidence. Clin Transl Allergy.

[16] Lombardi C, Bonini M, Passalacqua G, Mobile Apps Multicenter Italian Study Group (2018). The role of mobile apps in allergic respiratory diseases: an Italian multicentre survey report. Eur Ann Allergy Clin Immunol.

[17] Bousquet J, Farrell J, Crooks G, Hellings P, Bel EH, Bewick M (2016). Scaling up strategies of the chronic respiratory disease programme of the European Innovation Partnership on Active and Healthy Ageing (Action Plan B3: Area 5). Clin Transl Allergy.

[18] Bousquet J, Caimmi DP, Bedbrook A, Bewick M, Hellings PW, Devillier P (2017). Pilot study of mobile phone technology in allergic rhinitis in European countries: the MASK -rhinitis study. Allergy.

