# Supplementary material for: Public Prevention Plans to Manage Climate Change and Respiratory Allergic Diseases. Innovative Models Used in Campania Region (Italy): The Twinning Aria Implementation and the Allergy Safe Tree Decalogue
Source: Transl Med UniSa. 2019 Jan 6;19:95–102. (PMC6581484)
Supplement: Supplementary file 1 [file TM-19-095-s001.doc]

| BOX 1. ROLE OF MAIN ENVIRONMENTAL DETERMINANTS IN RELATION TO CLIMATE CHANGE |
| --- |
| - Increasing temperatures and extreme events: the increase in temperatures recorded in recent years causes the elongation and early arrival of the pollen season, an increase in pollen production, and the spread of invasive species. - Increase in the frequency of storms and intense rain: conditions of intense humidity and wind during thunderstorms can cause the breakage of pollen grains due to osmotic shock, promoting the release of allergenic antigens into the atmosphere (pollutant storms). Episodes of severe asthma during thunderstorms have been noted. - Increase in the frequency of fires and heat waves: in some regions, changes in the mean and variability of temperature and precipitation are expected to increase the frequency of fires and heat waves, with potential consequences on respiratory health. - Heat waves can aggravate pollution. Studies have shown that air pollution has an additional impact on mortality during a heat wave [6]. - High concentrations of ozone are the cause of excessive mortality due to respiratory issues during a heat wave [7]. - Changing weather conditions also influence the transportation, dispersion and depositing of atmospheric pollutants, and may impact the effect on health associated with fine powders and gaseous pollutants. - New atmospheric circulation models, caused by climatic variations, could also promote the long-distance transport of allergenic pollens, increasing the risk of new sensitisations among the allergic population [8]. - Effects on environmental air quality (indoor/outdoor) of marine aerosol currents may have an influence on the increase in respiratory illnesses in the general population. |
