# Supplementary material for: Public Prevention Plans to Manage Climate Change and Respiratory Allergic Diseases. Innovative Models Used in Campania Region (Italy): The Twinning Aria Implementation and the Allergy Safe Tree Decalogue
Source: Transl Med UniSa. 2019 Jan 6;19:95–102. (PMC6581484)
Supplement: Supplementary file 2 [file TM-19-095-s002.doc]

| BOX 2. CLIMATE CHANGE ALSO INFLUENCES THE EXPOSURE TO POLLUTANTS IN INDOOR AIR. CONDITIONS THAT MAY OCCUR DUE TO THERMAL (HEAT AND FROST WAVES) AND METEOROLOGICAL ANOMALIES |
| --- |
| - More time is spent indoors increasing the exposure period to indoor pollutants such as environmental tobacco smoke, benzene, NO2, PM and formaldehyde, which can cause an increase in the frequency of chronic respiratory symptoms, hyper-bronchial reactivity, and a reduced response to asthmatic therapy in asthmatic subjects. - Indoor micro-climatic alterations resulting from altered rainfall and temperature patterns have an influence on indoor biological pollutants (mould) which are important health risk factors when paired with humidity. - Excessive use of air conditioning during heat waves can lead to an increased exposure to microbiological contaminants which develop in the humid components of air conditioning systems. - Change in the ecosystem and allergenic determinants: current climate change is responsible for the early arrival of the spring pollen season, the prolongation of the flowering period and a change of the diffusion areas of main plant species. - The increase in the concentration of CO2 in the atmosphere and the increase in temperature (notably in the urban environment, where we are witnessing a phenomenon known as the “Urban Heat Island”, UHI) have encouraged the production of pollen and the development of certain allergenic plant species. |
