# Supplementary material for: Public Prevention Plans to Manage Climate Change and Respiratory Allergic Diseases. Innovative Models Used in Campania Region (Italy): The Twinning Aria Implementation and the Allergy Safe Tree Decalogue
Source: Transl Med UniSa. 2019 Jan 6;19:95–102. (PMC6581484)
Supplement: Supplementary file 3 [file TM-19-095-s003.doc]

| TABLE 1. PRIORITY ACTIONS TO MINIMISE RISKS ARISING FROM CLIMATE CHANGE, TO INCREASE THE RESILIENCE OF HUMAN AND NATURAL SYSTEMS AND THEIR EXPLOITATION, AND POTENTIAL OPPORTUNITIES ARISING FROM NEW CLIMATIC CONDITIONS. | |
| --- | --- |
| ACTION | OBJECTIVE OF THE ACTION |
| Implementation and strengthening of health monitoring and early alert systems, including monitoring systems on water-borne diseases. | Develop initiatives and adopting tools aimed at improving knowledge (of public health operators, physicians, and other professionals, including those in non-health sectors, institutions and citizens) on risk factors related to the environment and climate change, as well as on potential prevention strategies, with special attention to respiratory diseases such as asthma and allergies. |
| Training and refresher courses for operators of the SSN (Sistema Sanitario Nazionale, Italian national health system). | Contribute to promoting environmental prevention policies as a consequence of new risk scenarios triggered by climate change.  Improve scientific knowledge on the effects of climate change. |
| Strengthening of information and public communication systems. | Integrate institutions and scientific research in the health field, keeping the public opinion informed.  Disseminate the use of validated tools to support management and prevention of allergic rhinitis. |
